# High Rates of Non-Response Across Treatment Attempts in Chronic Irritable Bowel Syndrome: Results From a Follow-Up Study in Tertiary Care

**DOI:** 10.3389/fpsyt.2019.00714

**Published:** 2019-10-02

**Authors:** Yuanjun Dong, David Baumeister, Sabrina Berens, Wolfgang Eich, Jonas Tesarz

**Affiliations:** Department of General Internal Medicine and Psychosomatics, University Hospital Heidelberg, Heidelberg, Germany

**Keywords:** irritable bowel syndrome, treatment modalities, subjective experience, therapeutic impact, response rate

## Abstract

**Objective:** Despite a wealth of treatment options for irritable bowel syndrome (IBS), data on the subjective experience of treatments in ongoing clinical practice are sparse. This follow-up study assessed the individual usage of treatment modalities by IBS patients over time and investigated the patients’ subjective experience of therapeutic impact.

**Methods:** The study was conducted at the Specialty Clinic for Functional Gastrointestinal Disorders of the Heidelberg University Hospital. All patients who fulfilled the Rome III criteria for IBS and treated in our outpatient clinic between January 2012 and December 2016 were invited to the assessment. The primary outcome variables were individual usage of treatment modalities and the Patient Global Impression of Change (PGIC) with treatments.

**Results:** Three hundred and sixty-six patients fulfilled the Rome III criteria for IBS and thus were eligible for this study. Two hundred and seven patients dropped out from the study. The study could include 159 patients (43.7 ± 17.1 years; 71.1% female). The mean time since the first visit to the clinic was 2.8 ± 1.3 years (median 3.0 years). The mean time of symptom duration was 14.1 ± 11.1 years (median 10 years). The average number of treatment attempts was 12, ranging from 2 to 39). With respect to the subjective experience of therapeutic impact, there were no significant differences in the PGIC scores among different treatments (*p* = 0.183). The rates of non-response rates (minimally improved, no change, or minimally worse) ranged from 63.0% to 83.9%. The PGIC score was correlated negatively with the mean number of treatment attempts (*r* = −0.316, *p* < 0.01). The mean number of treatment attempts was correlated negatively with quality of life (*r* = −0.262, *p* < 0.01).

**Conclusion:** A multidisciplinary treatment approach of IBS is characterized by high rates of non-response and a high number of frustrating treatment attempts. The connection between the various treatment attempts and the frustrating subjective experience of therapeutic impact puts a substantial burden on IBS patients.

## Introduction

Irritable bowel syndrome (IBS) is a distressing chronic gastrointestinal disorder characterized by abdominal pain and changes in bowel habits (1). With a global prevalence of 9% to 12%, IBS is one of the most common functional gastrointestinal disorders in the world (2) and is associated with a substantial socioeconomic impact on the individual (3) as well as on society (4, 5). As the exact origin of IBS remains poorly understood, there are neither causal therapeutic approaches nor single-treatment interventions suitable and effective for all patients (6, 7). Accordingly, guidelines for the diagnosis and treatment of functional gastrointestinal diseases emphasize the combination of different therapies in a multimodal interdisciplinary treatment approach (8), including non-specific therapeutic recommendations (e.g., physical activity) as well as more specific recommendations, such as dietary advices, psychological interventions, and symptom-targeting medications. Although current guidelines (9) included a variety of different treatment options, adequate symptom control is still one of the greatest challenges in the treatment of IBS.

Against this background, a combination of several different treatment approaches is usually recommended in guidelines. However, Halder et al. (10) found that even after 10 years of treatment, patients with IBS are still plagued by various kinds of symptoms. In addition, more than half of IBS primary care counseling is due to patients being dissatisfied with previous treatments (11). Indeed, there is evidence that patients often use numerous treatments (12).

Despite a broad spectrum of IBS treatment options, few data have been published so far on the subjective experience of engagement with these treatment modalities and their performance under actual clinical conditions. While the superiority of several different treatment modalities over placebo was supported by a multitude of clinical trials (13), there is only limited evidence (14) for which treatments patients are engaged in and which are experienced as helpful by patients. Therefore, the aims of this study were 1) to determine the individual usage of treatment modalities by IBS patients over time and 2) to assess the patients’ subjective experience of therapeutic impact.

## Materials and Methods

### Study Design

This cross-sectional study was carried out at the Specialty Clinic for Functional Gastrointestinal Disorders at the Department of General Internal Medicine and Psychosomatics of Heidelberg University Hospital in tertiary care. This study was approved by the Ethics Committee of Heidelberg University (S-071/2017) and carried out in accordance with the Declaration of Helsinki and the Regulations for the Physicians of the Baden-Württemberg Chamber of Physicians in the latest versions. All patients who fulfilled the Rome III criteria for IBS and treated in our outpatient clinic between January 2012 and December 2016 were invited to the study. Patients received the study questionnaire package *via* mail, together with study invitation and consent forms, in October 2017. The study used the approach of the Dillman Total Design Method (15) to increase response rates.

**Figure 1:**
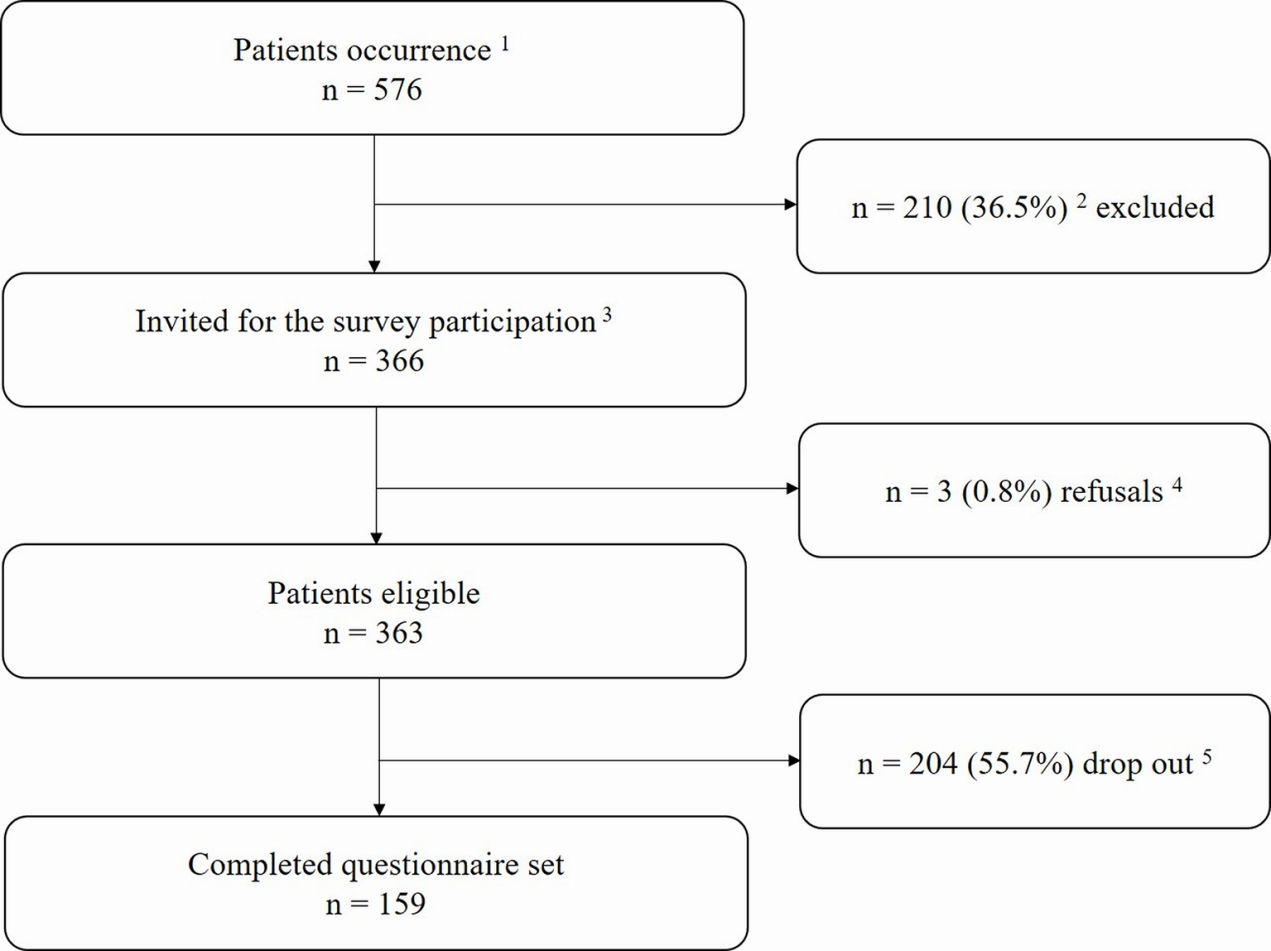
Flowchart of the study.^1^All patients treated within our functional gastrointestinal disorders (FGIDs) specialty care unit from January 2012 to December 2016 were screened for eligibility. ^2^Patients who did not meet the Rome III criteria for irritable bowel syndrome (IBS). ^3^The invitation period was from September 2017 to December 2017. ^4^Declared their refusal to participate by email or phone. ^5^Not available (e.g., missing contact data, did not respond).

There were 366 patients who fulfilled the Rome III criteria for IBS and thus were eligible for this study. Two hundred and four (55.7%) patients did not respond (including those whose new addresses were unknown), and three (0.8%) patients actively refused to participate in the study. Percentages of IBS subgroups, i.e., constipation predominant (IBS-C), diarrhea predominant (IBS-D), alternating or mixed (IBS-M), and undetermined (IBS-U), were also calculated in the patient cohort. The flowchart of patients’ responses and reasons for non-participation is shown in **Figure 1**.

***Inclusion/exclusion criteria*:** All patients had to be ≥18 years of age and had to provide signed informed consent. Patients were only included if they fulfilled the Rome III criteria for the diagnosis of IBS (16). Patients with illiteracy were excluded.

### Measures

In addition to the treatment modalities and the subjective experience of therapeutic impact, the sociodemographic data, symptom severity, psychological comorbidities, and quality of life were assessed by a set of general and functional gastrointestinal disorder–specific questionnaires.

#### Sociodemographic Data

Sociodemographic data including age, gender, family status, education level, duration of symptoms, and treatments were collected using the Psychosomatic Basis Documentation Questionnaire (Psy-BaDo) according to Heuft and Senf (17).

#### Symptomatic Characteristics

To assess the patients’ current symptomatic characteristics, symptom severity, quality of life, and psychological comorbidities were measured:

***Symptom severity*:** Symptom severity was evaluated using the IBS Symptom Severity Scale (IBS-SSS, range 0–500) (18, 19). High values indicate greater symptom burden, and the following cutoff values have been suggested: <75 healthy, 75–174 mild, 175–300 moderate, and >300 severe IBS (20).

***Quality of life*:** Quality of life was measured by the quality-of-life questionnaire for functional digestive disorders (FDDQL, range 0–100) (21, 22). FDDQL is a form of 48 items over eight domains (i.e., daily activity, disease-related anxiety, diet, sleep, discomfort, health perception, coping with disease, and impact of stress). Higher scores indicate better quality of life.

***Psychological comorbidities*:** Depression was measured using the nine-item depression module of the Patient Health Questionnaire (PHQ-9, range 0–27) (23). A cutoff value of ≥10 was interpreted as clinically relevant depressive comorbidity. Anxiety was assessed using the Generalized Anxiety Disorder seven-item questionnaire (GAD-7, range 0–21) (24). A cutoff value of ≥10 was used to indicate clinically relevant anxiety comorbidity. Disease-related fear was measured with the brief Whitley Index-7 (WI-7, range 0–28) (25). A cutoff value of >3 was interpreted as the presence of a clinically relevant level of disease-related fear.

#### Usage of Treatment Modalities and Subjective Experience of Therapeutic Impact

To explore the usage of treatment modalities and patients’ subjective experience of therapeutic impact, an additional questionnaire set was developed based on the German IBS treatment guidelines (9, 26) and the Patient Global Impression of Change (PGIC) scale (27).

To assess the individual usage of treatment modalities, participants were asked about their previous therapy experiences. Referring to the previous experience in this field (28), therefore, a structured and comprehensive list of different treatment modalities was developed. To develop this list, an initial focus group was employed. Next to the authors of the present work, the study involved 1) clinicians involved in the daily work with IBS patients, 2) clinical experts involved in the development of the German treatment guidelines for IBS, and 3) a methodologist. Based on the official German IBS guidelines (9, 26), an initial item pool was developed by this focus group, including all the treatment options generally recommended for IBS. This item pool was supplemented by various additional treatment options frequently reported by patients (e.g., complementary medicine, over-the-counter drugs). To assess the patients’ subjective experience of therapeutic impact for each treatment modality, we combined the treatment list with the seven-point Likert scale of the PGIC rating. Patients were asked to rate their subjective treatment satisfaction and global ratings of change of the overall situation using the following items: 1) very much improved, 2) much improved, 3) minimally improved, 4) no change, 5) minimally worse, 6) much worse, and 7) very much worse. Patients who rated PGIC with treatment as very much improved and much improved were treated as “improved”; minimally improved, no change, and minimally worse were treated as “non-response”; and very much worse and much worse were classified as “worsened” (29–31). After two rounds of piloting the comprehensibility, clarity, and comprehensiveness of this preliminary assessment, the treatment modalities were stratified according to five different treatment classes: non-specific general therapeutic recommendations (Cronbach’s α _non-specific general therapeutic recommendations_ = 0.582, e.g., physical activity, herbal tea, symptom diary); dietary recommendations (Cronbach’s α _dietary recommendations_ = 0.617, e.g., avoiding fructose, avoiding lactose, nutritional counseling); psychosocial interventions (Cronbach’s α _psychosocial interventions_ = 0.669, e.g., abdominal hypnotherapy, relaxation therapy, stress management); symptom-targeting medications (Cronbach’s α _symptom-targeting medications_ = 0.706, e.g., antidiarrhea drugs, antispasmodic drugs, acid inhibitor drugs); and complementary interventions (Cronbach’s α _complementary interventions_ = 0.847, e.g., homoeopathy, manual therapy, integrative mind–body therapy). The Cronbach’s α _overall_ coefficient in this study was 0.853.

### Statistical Analyses

All statistical analyses were performed using IBM SPSS Statistics 22.0 for Windows. Partial correlation was used to assess the relationship between the number of treatment attempts and the PGIC score. The average PGIC score for all treatments ever used was used in the analysis. Additionally, dropout analyses were performed to explore the impact of patients who completed the IBS diagnostic criteria at the initial visit but who dropped out in this study. For characterization of dropouts, data/medical records from the initial visit were used. All tests were two-sided. *P*-values less than 0.05 indicated statistical significance for all analyses. All analyses were explorative and not of a confirmatory nature; thus, no specific hypotheses were formulated.

## Results

### Sociodemographic and Symptomatic Characteristics

The study could include 159 (43.4%) patients (43.7 ± 17.1 years of age; 71.1% female). Of the patient cohort, 47.8% were classified as IBS-D, 43.4% were classified as IBS-M, and 6.3% were classified as IBS-C. As shown in **Table 1**, the mean time since the first visit to the clinic was 2.8 ± 1.3 years (median 3.0 years). The mean time of symptom duration was 14.1 ± 11.1 years (median 10.0 years). The mean level of symptom severity of these patients was 225.5 ± 101.8. Of all patients, 48.4% reported scores at moderate severity levels, 9.4% showed scores above the cutoff of value for severe symptom severity, and 32.1% showed scores at mild severity levels. Categorizing participants according to the validated cutoff values, the prevalence was 16.1% for depressive syndrome, 27.6% for anxiety syndrome, and 44.9% for disease-related fear. When considering the subgroups of IBS, there was no significant differences among the demographic and clinical characteristics, subjective experience of therapeutic impact, and number of treatment attempts between IBS-D and IBS-M. For more details, see **Table S1**.

**Table 1:** Demographic characteristics, symptom burden, and quality of life of the study cohort.

			IBS patients (n = 159)
Age		Mean ± SD (range)	43.4 ± 17.1 (18, 77)
Female		% (n)	71.1 (113)
Family status	Single	% (n)	47.4 (63)
	Stable cohabitation^1^		45.1 (60)
	Divorced or widowed		7.5 (10)
Education level above high school		% (n)	72.6 (106)
IBS subtypes	IBS-C	% (n)	6.3 (10)
	IBS-D		47.8 (76)
	IBS-M		43.4 (69)
Number of treatment attempts		Median (range)	12 (2, 39)
First onset of symptoms in years		Mean ± SD	14.1 ± 11.1
Clinic treatment period^2^ in years		Mean ± SD	2.8 ± 1.3
Symptom severity (IBS-SSS)		Mean ± SD	225.5 ± 101.8
Depression (PHQ-9)		Mean ± SD	5.4 ± 4.6
Anxiety (GAD-7)		Mean ± SD	6.7 ± 5.0
Disease-related fear (WI-7)		Mean ± SD	8.7 ± 6.5
Quality of life (FDDQL)		Mean ± SD	56.8 ± 11.2

^1^Stable cohabitation, i.e., married/unmarried cohabitation. ^2^Clinic treatment period: the mean period of time since the first visit in the specialty care outpatient clinic and follow-up assessment.

IBS, irritable bowel syndrome; IBS-C, IBS with constipation; IBS-D, IBS with diarrhea; IBS-M, IBS with mixed bowel habits; IBS-SSS, IBS Symptom Severity Scale; GAD-7, Generalized Anxiety Disorder seven-item questionnaire; PHQ-9, nine-item depression module of the Patient Health Questionnaire; WI-7, brief Whitley Index-7; FDDQL, quality-of-life questionnaire for functional digestive disorders; SD, standard deviation.

### Usage of Treatment Modalities and Subjective Experience of Therapeutic Impact

**Figure 2:**
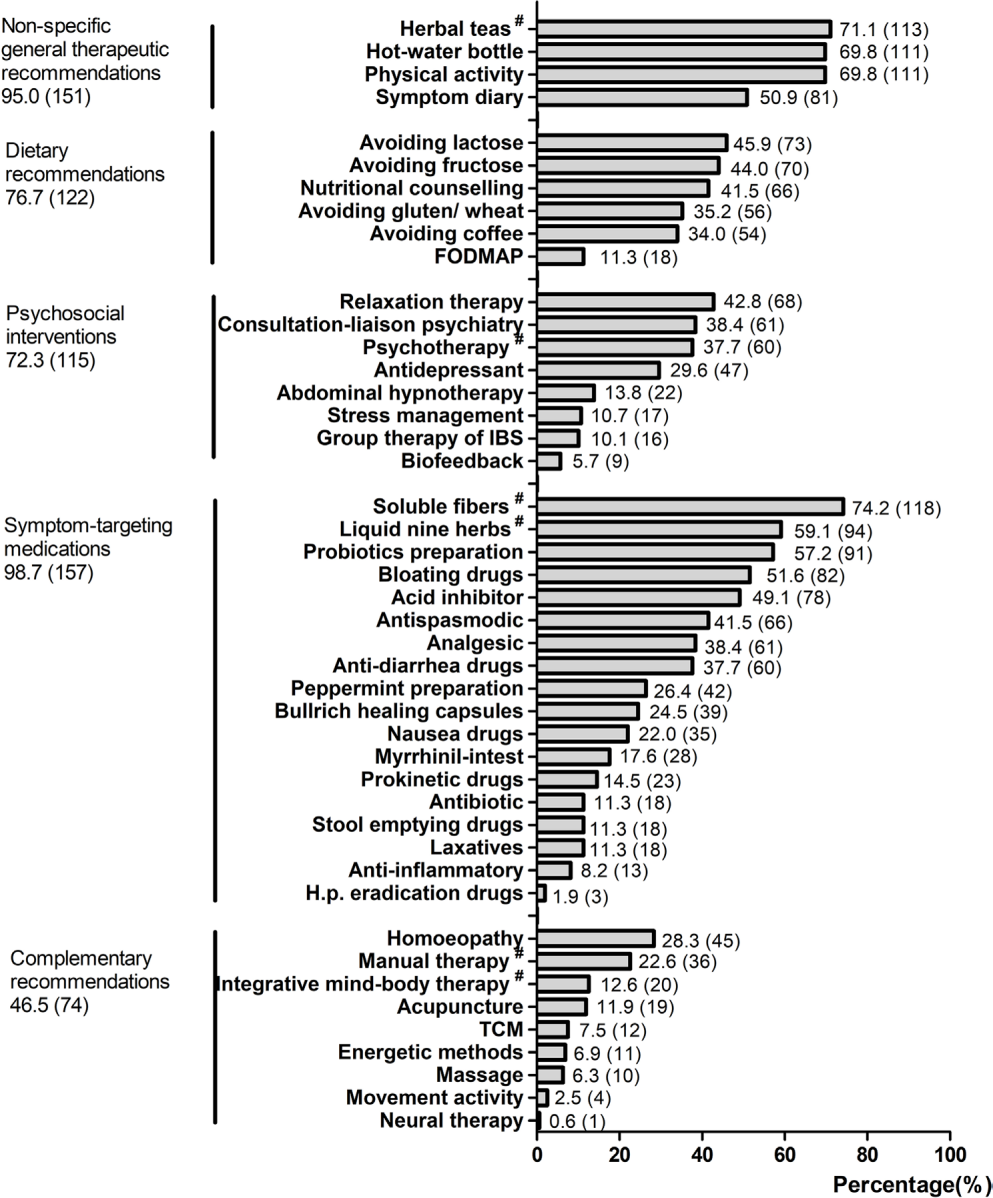
The usage of treatment modalities within each class. All values are shown as % (n). The set of five different treatment classes were based on the German IBS treatment guidelines and clinic practices; values represent the percentage of participants who reported previous treatment attempts with the treatment classes. FODMAP, fermentable oligo-, di-, mono-saccharides and polyols; TCM, traditional Chinese medicine. ^#^Herbal teas, e.g., fennel anise caraway tea; psychotherapy, e.g., cognitive behavioral therapy; soluble fibers, e.g., psyllium seed husks; liquid nine herbs, e.g., STW-5; manual therapy, e.g., osteopathy, chiropractic; integrative mind–body therapy, e.g., yoga, tai chi.

Patients reported on average experiences with treatments from at least two different treatment classes. The most-often-used treatment classes were symptom-targeting medications (98.7%, 157) and non-specific general therapeutic recommendations (95.0%, 151). The least-used class was complementary treatments (46.5%, 74). The average number of treatment attempts by patients was 12, ranging from 2 to 39. The five most-often-used treatment modalities were 1) soluble fibers (e.g., psyllium seed husks); 2) herbal teas (e.g., fennel anise caraway tea); 3) physical activity; 4) hot-water bottle; and 5) liquid nine herbs (e.g., STW-5). For more details of the usage of treatment modalities within each class, see **Figure 2**.

**Figure 3:**
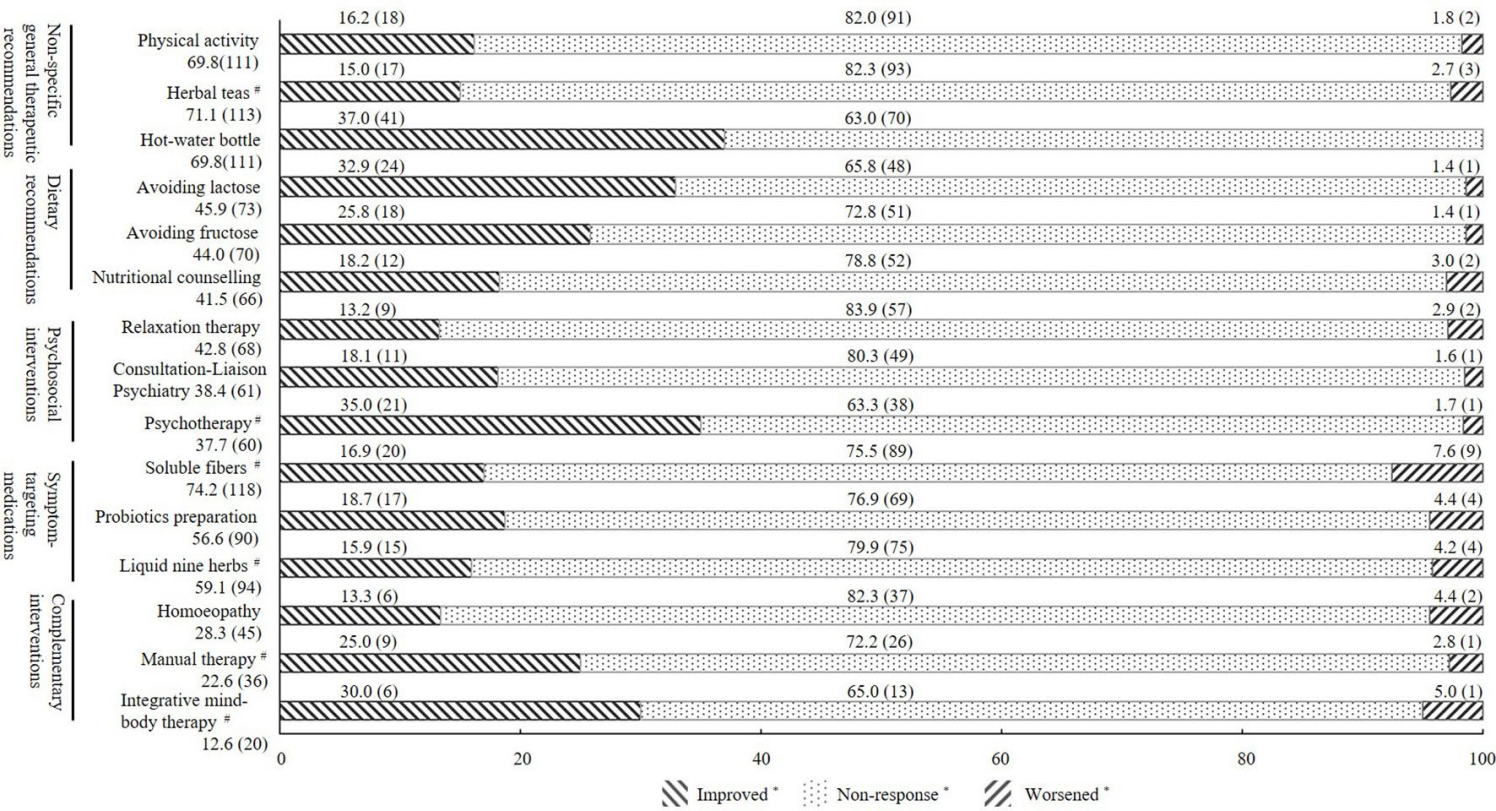
Subjective experience of therapeutic impact of the three most-often-used treatment modalities within each treatment class. All values are shown as % (n); *Improved, patients who rated Patient Global Impression of Change (PGIC) as very much improved or much improved; non-response, patients who rated PGIC as minimally improved, no change, or minimally worse; worsened, patients who rated PGIC as very much worse or much worse. ^#^Herbal teas, e.g., fennel anise caraway tea; psychotherapy, e.g., cognitive behavioral therapy; soluble fibers, e.g., psyllium seed husks; liquid nine herbs, e.g., STW-5; manual therapy, e.g., osteopathy, chiropractic; integrative mind–body therapy, e.g., yoga, tai chi.

With respect to the subjective experience of therapeutic impact, there were no significant differences in the PGIC scores among different treatments (*p* = 0.183). The rates of non-response (minimally improved, no change, or minimally worse) ranged from 63.0% to 83.9%. According to different treatment modalities, between 15% and 30% of all patients reported significant benefits (very much improved and much improved), and less than 5.0% reported that treatments have worsened their symptoms. For more details of the subjective experience of therapeutic impact within each class, see **Figure 3**. **Table S2** of the **Supplementary Material** presents the top five of the different treatment modalities stratified according to usage rate and subjective experience of therapeutic impact. **Figure S1** of the **Supplementary Material** presents the most-often-used treatments in general (treatments reported by <25% of the sample are not listed).

### Correlations Among the Subjective Experience of Impact, Number of Treatment Attempts, Symptom Severity, Psychological Comorbidities, and Quality of Life

The average PGIC score for all treatments ever used was used in the correlation analysis. Controlling the mean time of symptom duration and the mean period of time since the first visit, the PGIC score was correlated negatively with the mean number of treatment attempts (*r* = −0.320, *p* < 0.01). A similar relationship was found between PGIC score and the symptom severity (*r* = −0.381, *p* < 0.01). The mean number of treatment attempts also correlated negatively with quality of life (*r* = −0.263, *p* < 0.01). Depression, anxiety, and disease-related fear were all negatively correlated with PGIC score (*r* = −0.354, −0.279, −0.257, all *p* < 0.01). Meanwhile, depression and anxiety were both positively correlated with the number of attempted treatments (*r* = 0.184, 0.170, all *p* < 0.05). For more details, see **Table 2**.

**Table 2:** Partial correlation matrix among the subjective experience of therapeutic impact, number of treatment attempts, symptom severity, psychological comorbidities, and quality of life.

	1)	2)	3)	4)	5)	6)	7)
1) Overall PGIC^1^	1.000						
2) Treatment number^2^	−0.320**	1.000					
3) Symptom severity (IBS-SSS)	−0.381**	0.254**	1.000				
4) Depression (PHQ-9)	−0.354**	0.184*	0.461**	1.000			
5) Anxiety (GAD-7)	−0.279**	0.170*	0.504**	0.806**	1.000		
6) Disease-related fear (WI-7)	−0.257**	0.062	0.473**	0.552**	0.641**	1.000	
7) Quality of life (FDDQL)	0.427**	−0.263**	−0.725**	−0.472**	−0.558**	−0.584**	1.000
Sub-analyses							
PGIC number _non-specific general therapeutic recommendations_				−0.056			
PGIC number _dietary recommendations_				−0.212*			
PGIC number _psychosocial interventions_				0.012			
PGIC number _symptom-targeting medications_				−0.313**			
PGIC number _complementary interventions_				−0.261*			

*p < 0.05; **p < 0.01. ^1^Overall PGIC was used to measure the subjective experience of impact by all treatments ever used. ^2^Treatment number, i.e., number of attempted treatments ever used. Control variables were the duration of symptoms and the mean period of time since the first visit in the analyses. PGIC, Patient Global Impression of Change. IBS-SSS, IBS Symptom Severity Scale; GAD-7, Generalized Anxiety Disorder seven-item questionnaire; PHQ-9, nine-item depression module of the patient health questionnaire; WI-7, brief Whitley Index-7; FDDQL, quality of life questionnaire for functional digestive disorders.

### Dropout Analyses

Of the 207 dropouts, 67.1% (139) were female. The mean age was 36.8 ± 14.5 years (range 18–77 years). The study compared initial visit clinical questionnaire data between the participants who completed the study and those who dropped out. There were no statistically significant differences in regard to sociodemographic and symptomatic characteristics between participants and dropouts except the variables of age and IBS subtypes. For more details, see **Table S3** of the **Supplementary Material**.

## Discussion

### Findings

To our knowledge, this is the first study to evaluate specialty treatment practice variation among IBS patients at a tertiary care center in Germany. The study found that 1) IBS patients used an average of 12 different treatment modalities; 2) patients’ subjective experience of therapeutic impact (i.e., PGIC) scores with these treatments were characterized by high non-response rates, and there were no significant differences among different treatment modalities; and 3) the number of treatment attempts was negatively correlated with the subjective experience of therapeutic impact and the quality of life.

The most-often-used treatment classes in the study cohort were symptom-targeting medications (98.7%), such as soluble fibers (e.g., psyllium seed husks) and liquid nine herbs (e.g., STW-5), as well as non-specific general therapeutic recommendations (95.0%), such as physical activity and herbal teas (e.g., fennel anise caraway tea) for symptom reduction. This finding is in line with current guidelines, in which symptom-targeting medications are an important pillar for the treatment of IBS patients (9). As most patients suffer from more than one symptom, the use of numerous treatments is understandable. The treatments that patients in the study used are quite similar to those of a previous study from 2002 for the standard treatment of IBS (32). This study showed dietary advice, education, exercise advice, stress management and antispasmodic medications to be the most frequently used treatment modalities. This finding indicates that there have been no relevant changes in the medical care of IBS over the last 15 years.

In light of the unclear etiology of IBS and the resulting lack of causal therapies (33–35), it is not surprising that most patients use different treatment modalities. Accordingly, guidelines for the diagnosis and treatment of functional gastrointestinal diseases emphasize the combination of different therapies in a multimodal interdisciplinary treatment approach (8), including non-specific therapeutic recommendations as well as more specific recommendations, such as dietary advice, psychological interventions, and symptom-targeting medications. However, the number of treatment attempts with, on average, more than 12 different treatment modalities per patient was substantial in the study. In the face of this high number in combination with the high non-response rates, most treatments would be classified ineffective based on current clinical standards. Further, most treatment modalities were similar in terms of the perceived therapeutic impact. The rates of non-response were high in the study cohort, ranging on average from 63.0% to 83.9%. Most patients reported that previous treatments had hardly affected their symptoms so far. In line with this, almost two-thirds of the participants reported moderate to severe complaints and reduced quality of life despite multiple treatment attempts. These data are in agreement with those obtained by a French survey (36), which found that even though 87% of IBS patients reported using some form of medication, almost half of them considered their therapy to be ineffective. Similarly, a survey carried out at a large US health maintenance organization working in primary and secondary care found that a symptom reduction of more than 50% could be achieved only in approximately 22% of IBS patients (32). The study indicates that, at least with regard to the IBS patients seen at a tertiary IBS specialty clinic, no significant progress seems to have been achieved. One possibility is that IBS is a heterogeneous disorder in which clinical symptoms vary from person to person (37). What’s more, with varieties of symptoms and clinical features, IBS patients reflect many potential pathophysiological mechanisms (38). There were no significant relationships found when considering the correlation coefficient for PGIC number in non-specific general recommendations and psychosocial intervention. However, compared with other categories of treatment, psychosocial interventions and non-specific general recommendations are more susceptible to subjective conditions (e.g., cognition, personality, hypnosis), which means the responses to treatment vary more individually.

Although a high non-response rate was found, some mechanisms have also been reported. A possible mechanism to be discussed is the influence of previous negative treatment experiences on future therapy response. It is well known from nocebo research that negative expectations of a therapy have a strong potential to reduce future therapeutic effects (39). Given that the average IBS patient experiences a large number of frustrating therapy attempts, there is a risk that negative therapy expectations turn into a vicious cycle, with previous treatment failures leading to future treatment failures. Of note, anxiety was associated with an exacerbated nocebo response (40), and 44.9% of IBS patients in our study showed meaningful levels of disease-related fear, potentially indicating increased susceptibility to nocebo effects. However, these assumptions remain speculative, and further research is needed to better understand the underlying mechanisms.

### Limitations and Strengths

Several limitations of this study should also be considered. First, this was a single-center study in tertiary care; our findings therefore may not be representative of practice at other centers or hospitals but instead may reflect our clinic’s experience. The study cohort represents the outpatient patients, with higher disease burden and more psychological comorbidities than the primary care sample. Thus, the findings cannot be generalized. However, this long-term study shows what the current IBS patients are facing. Second, not all the subjects could be followed up, although we used repeated mailings to lower the dropout rate. However, our response rate is similar to those of other studies in this field (41–43). Moreover, dropout analyses did not indicate any obvious selection bias, at least concerning sociodemographic and symptomatic characteristics. This study used a retrospective design to measure the relationship between the usage of treatment modalities and the subjective experience of therapeutic impact. Therefore, there is a risk of a potential bias associated with self-report only. In addition to random measurement error, self-reports may be systematically biased if respondents have imperfect recall or deliberately provide misleading answers (44). The resolution of risks, which might generate spurious positive or null findings, requires large sample sizes in the future. Third, we did not assess the data of dose, duration, frequency, or order of therapies, as they may limit the efficacy to some extent. Using less effective treatments first may increase symptom severity and the opportunity for developing psychosocial distress. However, this research gap in the field of treatment still needs to be focused on in the future.

Despite those limitations, the strengths of this study should not be neglected. First, the IBS diagnosis was confirmed by a medical examination. Moreover, the treatment modalities were based on the German IBS treatment guidelines (9, 26). To capture the daily clinical perspectives in the best possible manner and to combine the scientific, clinical, and methodical experience, we used the method of focus group by including clinicians who work with IBS patients, clinical experts who are familiar with the German treatment guidelines for IBS, and a methodologist. It is well known that there is a high degree of variability among guidelines in the determination of need and type of IBS treatment in different countries. With this perspective, the findings on the subjective experience of therapeutic impact would be that IBS patients are often treated with therapies in clinic. Second, although there are many studies on efficacy and efficiency for a wide spectrum of different single treatment modalities, data on the patients’ subjective experience of therapeutic impact under actual clinical conditions in IBS are rather sparse (32, 45). In an attempt to close this gap, our results present data with a large sample size on individual treatment attempts and subjective experience of impact on a wide range of different treatment modalities embedded in an interdisciplinary tertiary care clinic of IBS treatment.

### Conclusions and Implications

To conclude, the multidisciplinary treatment approach of IBS is characterized by high rates of non-response and frustrating treatment attempts. Overall, IBS imposes a substantial burden on patients. This study demonstrates a complex treatment reality that is characterized by various treatment attempts and frustrating subjective experience of therapeutic impact.

## Data Availability Statement

The raw data supporting the conclusions of this manuscript will be made available by the authors, without undue reservation, to any qualified researcher. Requests for data should be sent to yuanjun.dong@med.uniheidelberg.de.

## Ethics Statement

All subjects gave written informed consent in accordance with the Declaration of Helsinki. The protocol was approved by the Ethics Committee of Heidelberg University (S-071/2017).

## Author Contributions

The study concept and design were made by YD and JT. Acquisition of data was carried out by YD. Statistical analysis and interpretation of data were done by YD, DB, and SB. Drafting of the paper was done by YD. Critical revision of the content was by JT, DB, WE, and SB. WE was responsible for administrative or material support. Study supervision was held by JT.

## Conflict of Interest

The authors declare that the research was conducted in the absence of any commercial or financial relationships that could be construed as a potential conflict of interest.

## Supplementary Material

Figure S1 Subjective experience of therapeutic impact of the most often used treatments in general. Treatments reported by <25% of the sample are not listed; All values are shown as % (n); *Improved, patients who rated PGIC as very much improved or much improved; Non-response, patients who rated PGIC as minimally improved, no change or minimally worse; Worsened, patients who rated PGIC as very much worse or much worse; ^#^Soluble fibres, e.g., psyllium seed husks; Herbal teas, e.g. fennel anise caraway tea; Liquid nine herbs, e.g., STW-5; Psychotherapy, e.g., cognitive behavioural therapy.

## References

[1] Canavan C West J Card T The epidemiology of irritable bowel syndrome. Clin Epidemiol (2014) 6:71–80. 10.2147/CLEP.S40245 24523597 PMC3921083

[2] Lovell RM Ford AC Global prevalence of and risk factors for irritable bowel syndrome: a meta-analysis. Clin Gastroenterol Hepatol (2012) 10:712–21. e714 10.1016/j.cgh.2012.02.029 22426087

[3] Agarwal N Spiegel BM The effect of irritable bowel syndrome on health-related quality of life and health care expenditures. Gastroenterol Clin (2011) 40:11–9. 10.1016/j.gtc.2010.12.013 21333898

[4] Canavan C West J Card T The economic impact of the irritable bowel syndrome. Aliment Pharmacol Ther (2014) 40:1023–34. 10.1111/apt.12938 25199904

[5] Longstreth GF Wilson A Knight K Wong J Chiou C Barghout V Irritable bowel syndrome, health care use, and costs: a US managed care perspective. Am J Gastroenterol (2003) 98:600–7. 10.1016/S0002-9270(02)06018-5 12650794

[6] Palsson OS Baggish JS Turner MJ Whitehead WE IBS patients show frequent fluctuations between loose/watery and hard/lumpy stools: implications for treatment. Am J Gastroenterol (2012) 107:286. 10.1038/ajg.2011.358 22068664 PMC3855407

[7] Hauser G Pletikosic S Tkalcic M Cognitive behavioral approach to understanding irritable bowel syndrome. World J Gastroenterol (2014) 20:6744. 10.3748/wjg.v20.i22.6744 24944466 PMC4051915

[8] Drossman DA Functional gastrointestinal disorders: history, pathophysiology, clinical features, and Rome IV. Gastroenterology (2016) 150:1262–79. e1262 10.1053/j.gastro.2016.02.032 27144617

[9] Layer P Andresen V Pehl C Allescher H Bischoof SC Classen M Irritable bowel syndrome: German consensus guidelines on definition, pathophysiology and management. Z. Gastroenterol (2011) 49:237. 10.3238/arztebl.2011.0751 21287438

[10] Halder SL Locke GR II Schleck CD Zinsmeister AR Melton LJ Talley NJ Natural history of functional gastrointestinal disorders: a 12-year longitudinal population-based study. Gastroenterology (2007) 133:799–807. 10.1053/j.gastro.2007.06.010 17678917

[11] Williams R Black C Kim HY Andrews EB Mangel AW Bud JJ Determinants of healthcare-seeking behaviour among subjects with irritable bowel syndrome. Aliment Pharmacol Ther (2006) 23:1667–75. 10.1111/j.1365-2036.2006.02928.x 16696818

[12] Camilleri M Di Lorenzo C The brain–gut axis: from basic understanding to treatment of irritable bowel syndrome and related disorders. J Pediatr Gastroenterol Nutr (2012) 54:446. 10.1097/MPG.0b013e31823d34c3 22027566 PMC3294167

[13] Ford AC Moayyedi P Lacy BE Lembo AJ Saito YA Schiller LR American College of Gastroenterology monograph on the management of irritable bowel syndrome and chronic idiopathic constipation. Am J Gastroenterol (2014) 109:S2. 10.1038/ajg.2014.187 25091148

[14] Usai-Satta P Bellini M Lai M Oppia F Cabras F Therapeutic approach for irritable bowel syndrome: old and new strategies. Curr Clin Pharmacol (2018)13:164–172. 10.2174/1574884713666180807143606 30084333

[15] Dillman DA The importance of adhering to details of the Total Design Method (TDM) for mail surveys. New Dir Prog Eval (1984) 21:49–64. 10.1002/ev.1359

[16] Drossman DA The functional gastrointestinal disorders and the Rome III process. Gastroenterology. (2006) 130:1377–90 10.1053/j.gastro.2006.03.008 16678553

[17] Heuft G Senf W Practice of quality management in psychotherapy: manual for the Psy-BaDo [psychotherapeutic basis documentation]. Stuttgart, Germany: Thieme (1998). 10.1007/s002780050099

[18] Francis CY Morris J Whorwell PJ The irritable bowel severity scoring system: a simple method of monitoring irritable bowel syndrome and its progress. Aliment Pharmacol Ther (1997) 11:395–402. 10.1046/j.1365-2036.1997.142318000.x 9146781

[19] Betz C Mannsdörfer K Bischoff S Validation of the IBS-SSS. Z. Gastroenterol (2013) 51:1171–6. 10.1055/s-0033-1335260 24122378

[20] Saigo T Tayama J Ogawa S Bernick PJ Takeoka A Hayashida M Increased risk of irritable bowel syndrome in university students due to gastrointestinal symptom–specific anxiety. Acta Medica Nagasakiensia (2018) 61:137–43. 10.11343/amn.61.137

[21] Chassany O Marquis P Scherrer B Read NW Finger T Bergmann JF Validation of a specific quality of life questionnaire for functional digestive disorders. Gut (1999) 44:527–33. 10.1136/gut.44.4.527 PMC1727453 10075960

[22] Yacavone RF Locke GR III Provenzale DT Eisen GM Quality of life measurement in gastroenterology: what is available? Am J Gastroenterol (2001) 96:285–97. 10.1111/j.1572-0241.2001.03509.x 11232666

[23] Kroenke K Spitzer RL Williams JB The PHQ-9: validity of a brief depression severity measure. J Gen Intern Med (2001) 16:606–13. 10.1046/j.1525-1497.2001.016009606.x PMC1495268 11556941

[24] Spitzer RL Kroenke K Williams GB Löwe B A brief measure for assessing generalized anxiety disorder: the GAD-7. Arch Intern Med (2006) 166:1092–7. 10.1001/archinte.166.10.1092 16717171

[25] Fink P Ewald H Jensen J Sørensen L Engberg M Holm M Screening for somatization and hypochondriasis in primary care and neurological in-patients: a seven-item scale for hypochondriasis and somatization. J Psychosom Res (1999) 46:261–73. 10.1016/S0022-3999(98)00092-0 10193917

[26] Hausteiner-Wiehle C Umgang mit Patienten mit nicht-spezifischen, funktionellen und somatoformen Körperbeschwerden: S3-Leitlinien mit Quellentexten, Praxismaterialien und Patientenleitlinie, Schattauer Verlag, 2013.

[27] Geisser ME Clauw DJ Strand V Gendreau RM Palmer R Williams D A. Contributions of change in clinical status parameters to Patient Global Impression of Change (PGIC) scores among persons with fibromyalgia treated with milnacipran. Pain ^®^ (2010) 149:373–8. 10.1016/j.pain.2010.02.043 20332060

[28] Berens S Kraus F Gauss A Tesarz J Herzog W Niesler B A specialty clinic for functional gastrointestinal disorders in tertiary care: concept and patient population. Clinical gastroenterology and hepatology. Off Clin Pract J Am Gastroenterol Assoc (2017) 15:1127. 10.1016/j.cgh.2017.02.039 28300691

[29] Middel B Stewart R Bouma J Sonderen E Heuvel WJA How to validate clinically important change in health-related functional status. Is the magnitude of the effect size consistently related to magnitude of change as indicated by a global question rating? J Eval Clin Pract (2001) 7:399–410. 10.1046/j.1365-2753.2001.00298.x 11737531

[30] Wyrwich KW Nienaber NA Tierney WM Wolinsky FD Linking clinical relevance and statistical significance in evaluating intra-individual changes in health-related quality of life. Med Care (1999) 37: 469–478. 10.1097/00005650-199905000-00006 10335749

[31] Farrar JT Young JP Jr LaMoreaux L Werth JL Poole RM Clinical importance of changes in chronic pain intensity measured on an 11-point numerical pain rating scale. Pain (2001) 94:149–58. 10.1016/S0304-3959(01)00349-9 11690728

[32] Whitehead WE Levy RL Von Korff M Feld AD Palsson OS Turner M The usual medical care for irritable bowel syndrome. Aliment Pharmacol Ther (2004) 20:1305–15. 10.1111/j.1365-2036.2004.02256.x 15606392

[33] Halland M Almazar A Lee R Atkinson E Larson J Talley NJ A case–control study of childhood trauma in the development of irritable bowel syndrome. Neurogastroenterol Motility (2014) 26:990–8. 10.1111/nmo.12353 24813232

[34] Barbara G Feinle-Bisset C Ghoshal UC Quigley EM Santos J Vanner S The intestinal microenvironment and functional gastrointestinal disorders. Gastroenterology (2016) 150:1305–18. 10.1053/j.gastro.2016.02.028 27144620

[35] Shanahan F Quigley EM Manipulation of the microbiota for treatment of IBS and IBD—challenges and controversies. Gastroenterology (2014) 146:1554–63. 10.1053/j.gastro.2014.01.050 24486051

[36] Dapoigny M Bellanger J Bonaz B Bruley des Varannes S Bueno L Coffin B Irritable bowel syndrome in France: a common, debilitating and costly disorder. Eur J Gastroenterol Hepatol (2004) 16:995–1001. 10.1097/00042737-200410000-00008 15371923

[37] Mujagic Z Ludidi S Keszthelyi D Hesselink MA Kruimel JW Lenaerts K Small intestinal permeability is increased in diarrhoea predominant IBS, while alterations in gastroduodenal permeability in all IBS subtypes are largely attributable to confounders. Aliment Pharmacol Ther (2014) 40:288–97. 10.1111/apt.12829 24943095

[38] Gazouli M Wouters MM Kapur-Pojskić L Bengtson MB Friedman E Nikčević G Lessons learned—resolving the enigma of genetic factors in IBS. Nat Rev Gastroenterol Hepatol (2016) 13:77. 10.1038/nrgastro.2015.206 26726033

[39] Enck P Benedetti F Schedlowski M New insights into the placebo and nocebo responses. Neuron (2008) 59:195–206. 10.1016/j.neuron.2008.06.030 18667148

[40] Staats PS Staats A Hekmat H The additive impact of anxiety and a placebo on pain. Pain Med (2001) 2:267–79. 10.1046/j.1526-4637.2001.01046.x 15102231

[41] Zernicke KA Campbell TS Blustein PK Fung TS Johnson JA Bacon SL Mindfulness-based stress reduction for the treatment of irritable bowel syndrome symptoms: a randomized wait-list controlled trial. Int J Behav Med (2013) 20:385–96. 10.1007/s12529-012-9241-6 22618308

[42] Abdul-Baki H El Hajj II Elzahabi L Azar C Aoun E Skoury A A randomized controlled trial of imipramine in patients with irritable bowel syndrome. World J Gastroenterol (2009) 15:3636. 10.3748/wjg.15.3636 19653341 PMC2721237

[43] Mazzawi T Hausken T Gundersen D El-Salhy M Effect of dietary management on the gastric endocrine cells in patients with irritable bowel syndrome. Eur J Clin Nutr (2015) 69:519. 10.1038/ejcn.2014.151 25097003 PMC4387551

[44] Bauhoff S Systematic self-report bias in health data: impact on estimating cross-sectional and treatment effects. Health Serv Outcomes Res Methodol (2011) 11:44–53. 10.1007/s10742-011-0069-3

[45] Törnblom H Goosey R Wiseman G Baker S Emmanuel A Understanding symptom burden and attitudes to irritable bowel syndrome with diarrhoea: results from patient and healthcare professional surveys. United Eur Gastroenterol J (2018) 6:1417–27. 10.1177/2050640618787648 PMC6206540 30386615

