# Supplementary figures and images for: High Rates of Non-Response Across Treatment Attempts in Chronic Irritable Bowel Syndrome: Results From a Follow-Up Study in Tertiary Care
Source: Front Psychiatry. 2019 Oct 2;10:714. doi: 10.3389/fpsyt.2019.00714 (PMC6797829; doi:10.3389/fpsyt.2019.00714)

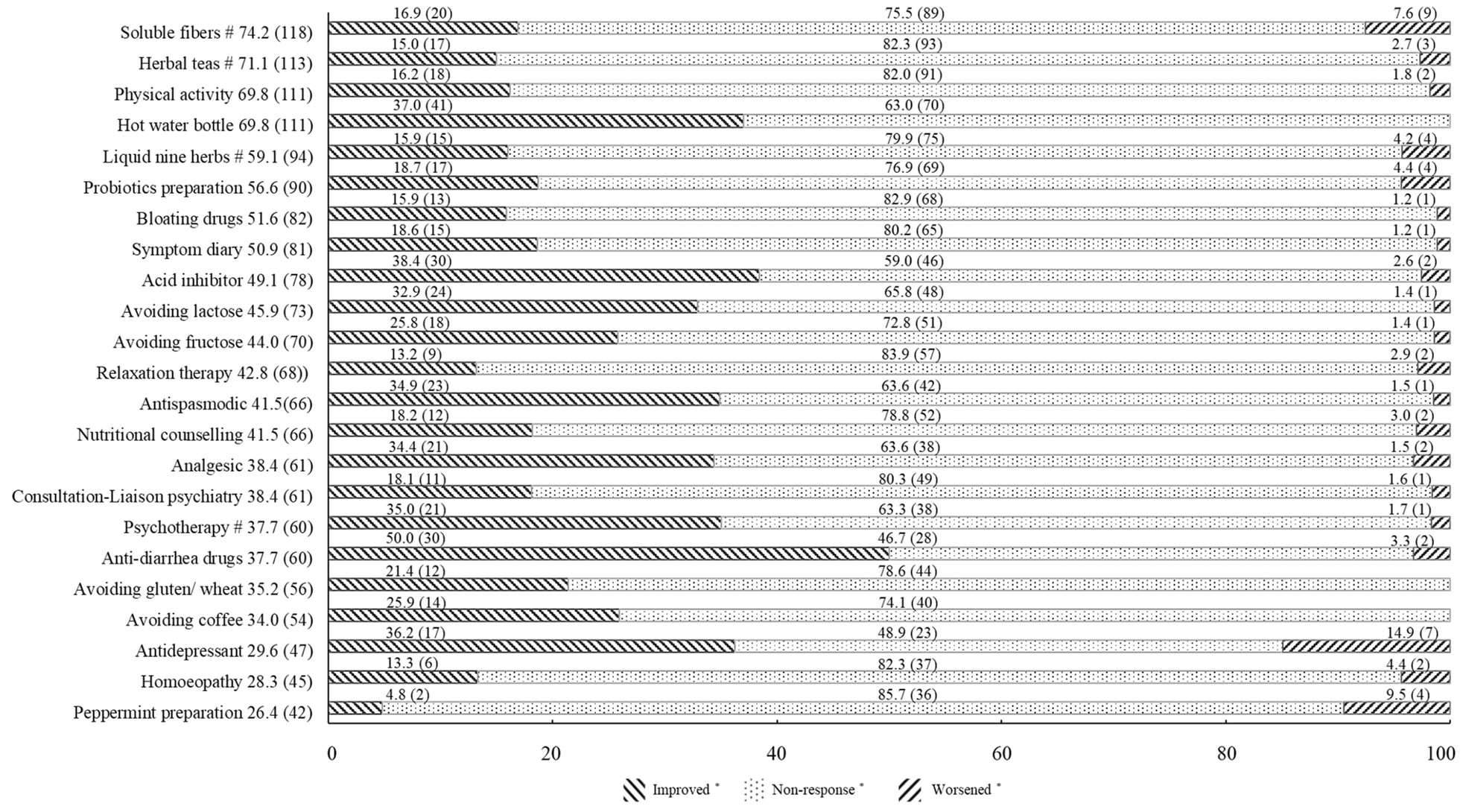

Supplement: Figure S1 — Subjective experience of therapeutic impact of the most often used treatments in general. Treatments reported by <25% of the sample are not listed; All values are shown as % (n); *Improved, patients who rated PGIC as very much improved or much improved; Non-response, patients who rated PGIC as minimally improved, no change or minimally worse; Worsened, patients who rated PGIC as very much worse or much worse; #Soluble fibres, e.g., psyllium seed husks; Herbal teas, e.g. fennel anise caraway tea; Liquid nine herbs, e.g., STW-5; Psychotherapy, e.g., cognitive behavioural therapy. [file Image_1.jpeg]
