# Supplementary material for: High Rates of Non-Response Across Treatment Attempts in Chronic Irritable Bowel Syndrome: Results From a Follow-Up Study in Tertiary Care
Source: Front Psychiatry. 2019 Oct 2;10:714. doi: 10.3389/fpsyt.2019.00714 (PMC6797829; doi:10.3389/fpsyt.2019.00714)
Supplement: Supplementary file 2 [file DataSheet_1.docx]

**Supplementary Tables**

**Table S1.** **Comparison for the demographic and clinical characteristics, subjective experience of therapeutic impact and number of treatment attempts between IBS-D and IBS-M**

|  | | IBS-D  n=76 | IBS-M  n=69 | *t or χ2* | *p* |
| --- | --- | --- | --- | --- | --- |
| Age | | 43.5±17.6 | 41.2±16.2 | 0.833 | 0.406 |
| Female | | 65.8(50) | 72.5(50) | 0.753 | 0.386 |
| Family status | Single | 49.3(37) | 44.9(31) | 0.741 | 0.690 |
|  | Stable cohabitation ^1^ | 42.7(32) | 49.3(34) |  |  |
|  | Divorced or widowed | 8.0(6) | 5.8(4) |  |  |
| Education level above high school | | 72.0(54) | 76.5(52) | 0.372 | 0.542 |
| First onset of symptoms in years | | 13.4±10.0 | 14.7±12.2 | -0.666 | 0.507 |
| Clinic treatment period ^2^ in years | | 2.8±1.3 | 2.7±1.3 | 0.567 | 0.571 |
| Overall number of treatment attempts | | 12.6±5.6 | 13.9±7.1 | -1.246 | 0.215 |
| Number _Non-specific general therapeutic recommendations_ | | 2.5±1.2 | 2.8±1.1 | -1.540 | 0.126 |
| Number _Dietary recommendations_ | | 2.0±1.6 | 2.2±1.8 | -0.808 | 0.420 |
| Number _Psychosocial interventions_ | | 1.7±1.6 | 2.1±1.9 | -1.384 | 0.168 |
| Number _Symptom-targeting medications_ | | 5.6±2.9 | 5.9±3.4 | -0.579 | 0.563 |
| Number _Complementary interventions_ | | 0.9±1.3 | 1.0±1.5 | -0.284 | 0.777 |
| Overall PGIC score ^3^ | | 3.1±0.6 | 3.1±0.7 | -0.407 | 0.685 |
| PGIC _Non-specific general therapeutic recommendations_ | | 3.2±0.7 | 3.1±0.7 | 1.042 | 0.299 |
| PGIC _Dietary recommendations_ | | 3.0±0.8 | 3.1±0.9 | -0.962 | 0.338 |
| PGIC _Psychosocial interventions_ | | 3.3±1.1 | 3.3±1.1 | -0.223 | 0.824 |
| PGIC _Symptom-targeting medications_ | | 3.1±0.7 | 3.1±0.8 | -0.122 | 0.903 |
| PGIC _Complementary interventions_ | | 3.2±0.8 | 3.3±1.1 | -0.702 | 0.485 |
| Symptom severity (IBS-SSS) | | 222.3±106.2 | 229.9±95.1 | -0.455 | 0.650 |
| Depression (PHQ-9) | | 5.5±4.9 | 5.4±3.8 | 0.165 | 0.870 |
| Anxiety (GAD-7) | | 6.5±4.9 | 6.9±4.8 | -0.590 | 0.556 |
| Disease-related fear (WI-7) | | 10.3±5.7 | 11.1±5.8 | -0.769 | 0.443 |
| Quality of life (FDDQL) | | 57.5±11.6 | 56.6±10.2 | 0.481 | 0.632 |

***Note:*** *^1^ Stable cohabitation, i.e., married/ unmarried cohabitation; ^2^ Clinic treatment period: the mean period of time since first visit in the specialty care outpatient clinic and follow-up assessment. ^3^ Overall PGIC was used to measure the subjective experience of impact by all treatments ever used; All values are shown as Mean±SD or % (n); IBS-SSS ranges from 0 to 500; PHQ-9 ranges from 0 to 27; GAD-7 ranges from 0 to 21; WI-7 ranges from 0 to 28; FDDQL ranges from 0 to 100.* ***Abbreviation:*** *IBS-SSS, IBS Symptom Severity Scale; GAD-7, Generalized Anxiety Disorder seven-item questionnaire; PHQ-9, nine-item depression module of the patient health questionnaire; WI-7, brief Whitley Index-7; FDDQL, quality of life questionnaire for functional digestive disorders; SD, standard deviation.*

**Table S2. Rankings (“Top 5”) of different treatment modalities stratified according to usage rate and subjective experience of therapeutic impact**

|  | Most often used | | Improved ^*^ | | Non-response ^*^ | | Worsened ^*^ | |
| --- | --- | --- | --- | --- | --- | --- | --- | --- |
|  | treatment | usage rate | treatment | improved rate | treatment | non-response rate | treatment | worsened rate |
| 1) | Soluble fibers ^#^ | 74.2 (118) | Antidiarrhea drugs ^#^ | 50.0 (30) | Mucosal healing Capsule ^#^ | 87.2 (34) | Antidepressant drugs ^#^ | 14.9 (7) |
| 2) | Herbal teas^#^ | 71.1 (113) | Nausea drugs^#^ | 45.7 (16) | Peppermint preparation | 85.7 (36) | Peppermint preparation | 9.5 (4) |
| 3) | Physical activity | 69.8 (111) | Acid inhibitor^#^ | 38.5 (30) | Relaxation techniques | 83.8 (57) | Soluble fibers^#^ | 7.6 (9) |
| 4) | Hot water bottle | 69.8 (111) | Hot-water bottle | 36.9 (41) | Bloating drugs^#^ | 82.9 (68) | Homoeopathy | 4.4 (2) |
| 5) | Liquid nine herbs ^#^ | 59.1 (94) | Antidepressant drugs ^#^ | 36.2 (17) | Herbal teas^#^ | 82.3 (93) | Probiotics preparation | 4.4 (4) |

***Note:*** *All values are shown as % (n); ^*^ Improved, patients who rated global impression of change with treatment as very much improved or much improved; Non-response, patients who rated global impression of change with treatment as minimally improved, no change or minimally worse; Worsened, patients who rated global impression of change with treatment as very much worse or much worse. ^#^ Soluble fibers: e.g., psyllium seed husks; Herbal teas: e.g., fennel anise caraway tea; Liquid nine herbs: e.g., STW-5; Antidiarrhea drugs: e.g., loperamide; Nausea drugs: e.g., dimenhydrinat; Acid inhibitor: e.g., pantoprazol; Antidepressant drugs: e.g., sertralin; Mucosal healing Capsule: e.g., ketotifen; Bloating drugs: e.g., simeticon.*

**Table S3. Comparison for the initial visit clinical feature between participants and drop outs**

|  |  | Participates  *n*=159 | Drop outs  *n*=207 | *t or χ2* | *p* |
| --- | --- | --- | --- | --- | --- |
| Age | | 43.4±17.1 | 36.8±14.5 | 3.977 | <0.001 |
| Female |  | 71.0% (110) | 67.1% (139) | 0.602 | 0.438 |
| Family status | Single | 47.7% (62) | 41.4% (75) | 1.206 | 0.547 |
|  | Stable cohabitation ^1^ | 44.6% (58) | 49.7% (90) |  |  |
|  | Divorced or widowed | 7.7% (10) | 8.8% (16) |  |  |
| Education level above high school | | 72.5% (103) | 69.4% (127) | 0.380 | 0.537 |
| IBS subtypes | IBS-C | 6.6 (10) | 13.0% (26) | 6.574 | 0.037 |
|  | IBS-D | 49.0% (74) | 37.5% (75) |  |  |
|  | IBS-M | 44.4% (67) | 49.5% (99) |  |  |
| Symptom severity (IBS-SSS) | | 267.6±83.2 | 282.0±92.2 | -0.873 | 0.383 |
| Anxiety (GAD-7) | | 6.9±4.9 | 7.7±5.4 | -0.980 | 0.328 |
| Depression (PHQ-9) | | 8.9±5.5 | 7.8±5.4 | -1.619 | 0.106 |
| Disease-related fear (WI-7) | | 10.8±5.8 | 11.7±6.8 | -1.100 | 0.272 |
| Quality of life (FDDQL) | | 49.7±13.3 | 48.5±13.6 | 0.778 | 0.437 |

***Note:*** *^1^ Stable cohabitation, i.e., married/ unmarried cohabitation; All values are shown as Mean±SD or % (n); IBS-SSS ranges from 0 to 500; PHQ-9 ranges from 0 to 27; GAD-7 ranges from 0 to 21; WI-7 ranges from 0 to 28; FDDQL ranges from 0 to 100.* ***Abbreviation:*** *IBS, irritable bowel syndrome; IBS-C, IBS with constipation; IBS-D, IBS with diarrhea; IBS-M, IBS with mixed bowel habits; IBS-SSS, IBS Symptom Severity Scale; GAD-7, Generalized Anxiety Disorder seven-item questionnaire; PHQ-9, nine-item depression module of the patient health questionnaire; WI-7, brief Whitley Index-7; FDDQL, quality of life questionnaire for functional digestive disorders; SD, standard deviation.*
